# EGF Receptor-Targeted Synthetic Double-Stranded RNA Eliminates Glioblastoma, Breast Cancer, and Adenocarcinoma Tumors in Mice

**DOI:** 10.1371/journal.pmed.0030006

**Published:** 2005-12-06

**Authors:** Alexei Shir, Manfred Ogris, Ernst Wagner, Alexander Levitzki

**Affiliations:** 1Department of Biological Chemistry, The Hebrew University of Jerusalem, Givat Ram, Jerusalem, Israel; **2**Pharmaceutical Biology-Biotechnology, Department of Pharmacy, Ludwig-Maximilians-Universität, Munich, Germany; Genome Institute of SingaporeSingapore

## Abstract

**Background:**

Glioblastoma multiforme (GBM) is the most lethal form of brain cancer. With the available treatments, survival does not exceed 12–14 mo from the time of diagnosis. We describe a novel strategy to selectively induce the death of glioblastoma cells and other cancer cells that over-express the EGF receptor. Using a non-viral delivery vector that homes to the EGF receptor, we target synthetic anti-proliferative dsRNA (polyinosine-cytosine [poly IC]), a strong activator of apoptosis, selectively to cancer cells.

**Methods and Findings:**

Poly IC was delivered by means of a non-viral vector: 25kDa polyethylenimine-polyethyleneglycol-EGF (PEI_25_-PEG-EGF). EGFR-targeted poly IC induced rapid apoptosis in the target cells in vitro and in vivo. Expression of several cytokines and “bystander killing” of untransfected tumor cells was detected in vitro and in vivo. Intra-tumoral delivery of the EGFR-targeted poly IC induced the complete regression of pre-established intracranial tumors in nude mice, with no obvious adverse toxic effects on normal brain tissue. A year after treatment completion the treated mice remain cancer-free and healthy. Similarly, non-viral delivery of poly IC completely eliminated pre-established breast cancer and adenocarcinoma xenografts derived from EGFR over-expressing cancer cell lines, suggesting that the strategy is applicable to other EGFR-over-expressing tumors.

**Conclusion:**

The strategy described has yielded an effective treatment of EGFR over-expressing GBM in an animal model. If this strategy is translated successfully to the clinical setting, it may actually offer help to GBM patients. Moreover the elimination of two additional EGFR over-expressing cancers in vivo suggests that in principle this strategy can be applied to treat other tumors that over-express EGFR.

## Abstract 2

A non-viral vector delivers double-stranded RNA predominantly to tumor cells overexpressing EGFR. This leads to apoptosis in tumor cells and eliminates established tumors in mice.

## Introduction

Glioblastoma multiforme (GBM), a brain cancer, is one of the deadliest human diseases, and cannot be cured by any therapy available today. The localization of GBM in the central nervous system and the very solid structure of this tumor renders it almost impermeable to large particles, such as viral vectors [1]. A major challenge in the treatment of GBM is to kill the accessible cancer cells on the surface of the tumor more rapidly than the rate of replication of the cells. Otherwise, the unexposed, internal cells can replicate and compensate for the cells that have just been eliminated. Thus, an effective treatment for GBM must incorporate the following features: (a) high selectivity and safety, to avoid damage to non-cancerous brain tissue; (b) rapid and efficient cell killing, preferably by simultaneous activation of multiple killing mechanisms. The simultaneous activation of multiple killing pathways will ensure tumor cell death, even if one or several pathways are inactive; and, (c) inhibition of the growth or killing of neighboring, unexposed tumor cells. This “bystander effect” should assist in eliminating the tumor before it can re-grow. It should also inhibit the growth of any tumor cells that may have a different phenotype from the targeted cells and are not themselves targeted by the treatment, including cancer stem cells.

In an attempt to meet all these demands in one treatment, we have taken advantage of the frequent (50%–70%) over-expression of epidermal growth factor receptor (EGFR) in GBM [2]. We have attached synthetic, double-stranded RNA (dsRNA) to a non-viral vector that can home in on EGFR. The dsRNA is selectively introduced into the cancer cells via receptor-mediated endocytosis. Double-stranded RNA, frequently expressed in cells infected with viruses, activates a number of pro-apoptotic processes simultaneously. These include the dsRNA dependent protein kinase (PKR) and 2,5- oligo-A synthetase, both of which turn off protein synthesis [3]. Double-stranded RNA also activates p38 and JNK, and stimulates the synthesis of pro-apoptotic proteins, such as IRF3-DRAF1 and NFκB [3–5]. These dsRNA-induced mechanisms efficiently kill infected cells and induce expression of anti-proliferative cytokines from the interferon family, thereby preventing spread of the virus [4].

In order to specifically introduce poly IC into EGFR over-expressing cells, we utilized polyethylenimine (25 kDa)-polyethylene-glycol-mEGF (PEI_25_-PEG-EGF) complexes [6,7]. We expected this approach to be highly selective, because the number of EGFRs on tumor cells is 10–100 times higher than that on non-tumor cells [2]. PEI_25_-PEG-EGF conjugates are significantly safer than replication-deficient or replication-competent viruses, in terms of immunotoxic reactions, inadvertent recombination and viral replication in healthy cells. Cell death was expected to be fast, because dsRNA activates cell killing mechanisms within minutes of entering the cell. Finally, induction of interferons, clinically used against GBM, was expected to exert a bystander effect and inhibit the growth of adjacent, untransfected tumor cells.

## Methods

### Reagents and Assays

Poly IC was obtained from Sigma (Rehovot, Israel). It was dissolved in DEPC-treated double-distilled H_2_O. The polyethylenimine (PEI), PEI25, branched and succinimidyl 3-(2-pyridyldithio) propionate (SPDP) were purchased from Sigma-Aldrich (Munich, Germany). NHS-PEG-MAL (MW = 3400) was obtained from Nektar Therapeutics (Huntsville, Alabama, United States) and the recombinant mouse EGF (mEGF) from Pepro Tech EC Ltd. (London, United Kingdom). The PEI content of the conjugate was determined spectrophotometrically by TNBS assay at 405 nm. The amount of dithiopyridine linkers in PEI was determined after reduction of an aliquot with dithiothreitol (DTT) followed by absorption measurement of released pyridine-2-thione at 343 nm.

The molar ratio of mEGF: dithiopyridine was determined spectrophotometrically at 280 and 340 nm. The amount of dithiopyridine was determined as described [6,7]. The yield of mEGF (mg) was calculated in two equations. Equation 1: A_280 (a)_ = A_340_ with DTT × 5.1/8.1. Equation 2: A_280_ revised = A_280_−A_280 (a)_. The result of equation 2 was the amount of mEGF in mg.

The Ellman assay was used for the determination of the mercapto groups in mEGF-SH. Liquid chromatography of conjugates was performed with the ÄKTA basic system from Amersham Biosciences (Little Chalfont, United Kingdom).

Melittin (Mel) (D-Mel-SH; _280_ = 5570, MW = 2893.6) was purchased from IRIS Biotech GmbH (Marktredwitz, Germany).

All other chemicals were purchased from Sigma-Aldrich.

### Fluorescence Microscopy

Poly IC was labeled with the Fluorescein ULS labeling kit (Fermentas, Hanover, Maryland, United States) at 1 unit of fluorescein per 1 μg of poly IC according to the manufacturer's instructions and then condensed with the appropriate PEI conjugate. Cells were incubated with the complexes in DMEM/FCS for 4 h at 37 °C and washed twice with PBS. Cells were viewed on a Zeiss confocal microscope. Green fluorescence was viewed with filter sets appropriate for fluorescein.

### In Vitro Apoptosis Detection

Cells were seeded into 24-well plates at a density of 10,000 in 1 ml of medium per well and grown overnight. After appropriate treatment cells were washed, fixed and stained using the Annexin-V-Biotin kit (Roche, Basel, Switzerland) according to the manufacturer's instructions. In addition apoptotic death was also determined by TUNEL assay using the In situ Cell Death Detection kit (Roche). The brown colored apoptotic cells were visualized in a microscope, counted (6 fields per sample), and photographed using digital camera.

### In Vitro Bystander Effect

For this assay, 500,000 U87MGwtEGFR cells were seeded onto 6-cm plates, grown overnight in 2 ml of medium, and transfected with 1 μg/ml poly IC using PEI-PEG-EGF+PEI-Mel complexes. Medium was collected at 24 h after transfection. U87MG and U87MGΔEGFR “indicator” cells were seeded in duplicate in 96-well plates (4,000 cells/well) and grown overnight in 200 μl of medium. Then, 100 μl of medium was then replaced by the medium collected from the transfected (**+poly IC**) or untransfected (**−poly IC**) U87MGwtEGFR cells. Where indicated the medium was pre-incubated for 1 h at room temperature, with neutralizing polyclonal anti-IFN-α antibody (1:500, Santa Cruz Biotechnology, Santa Cruz, California, United States). In **NT** samples, medium was not replaced. Growth inhibition was examined 48 h after medium exchange.

### Effect of Targeted Poly IC on 10-d-Old Intracranial Tumor Models

For this assay, 10,000 U87MGwtEGFR cells per animal were stereotactically implanted into the brains of 35 nude mice as described [8]. Five animals were sacrificed 10 d later to evaluate the sizes of the tumors [8]. Then, 200 μl of Alzet (Alzet, Cupertino, California, United States) osmotic micropumps with intra-tumoral catheters were installed in 20 mice. Ten mice received (poly IC)PEI-PEG-EGF+PEI-Mel complex dissolved in HBG buffer [9] at 0.1 μg poly IC/μl buffer (the dose of poly IC was 0.8 μg/h or 19.2 μg/d), and ten others received equivalent doses of PEI-PEG-EGF+PEI-Mel complexes only (no poly IC). Ten remaining control animals did not receive any treatment. The pumps were replaced twice every 24 h. At day 20 after cell implantation (10 d after treatment initiation), five animals from each group were sacrificed to evaluate tumor size as described [8]. Five other animals in each group were retained for survival analysis [8]. All animal experiments were conducted in accordance with the Hebrew University guidelines for the care of laboratory animals.

### In Vivo Bystander Effect

For this assay, 5,000 U87MGwtEGFR cells were mixed with 5,000 U87MGΔEGFR cells in 5 μl of PBS per animal. The cells were stereotactically implanted into the brains of 14 nude mice as described [8]. 10 d later, 200-μl Alzet osmotic micropumps with intra-tumoral catheters were installed in 12 mice. Six mice received (poly IC)PEI-PEG-EGF+PEI-Mel (**poly IC/conjugate)** complexes dissolved in HBG buffer at the indicated dose (two animals for each dose), and six others received an equivalent dose of PEI-PEG-EGF+PEI-Mel complexes without poly IC (**Conjugate only**). The pumps were replaced twice every 24 h. Survival of the animals was analyzed as above.

### In Vivo Apoptosis Detection

For this assay, 10,000 U87MGwtEGFR cells per animal were stereotactically implanted into the brains of 12 mice as above. 14 d later, 200-μl Alzet osmotic micropumps with intra-tumoral catheters were installed in six U87MGwtEGFR-bearing mice. Three animals received formulated poly IC (19.2 μg total per animal for 24 h), and three mice received complexes without poly IC. Six remaining animals received no treatment. Immediately after termination of infusion (15 d after cell implantation), animals were sacrificed, the brains were fixed with 4% formalin, embedded in paraffin, and ultra-thin slices were prepared. Slices were then de-paraffinized and analyzed by fluorescent immunohistochemistry to determine apoptosis with the Cell Death Detection kit-TMR Red, Roche (red fluorescence) and EGFR expression with FITC conjugated EGFR (internal domain) antibody (green fluorescence), Biosource (Camarillo, California, United States).

### Synthesis of Mel-PEI_25_-PEG-mEGF

The synthesis of mEGF-PEG-PEI25 is described elsewhere [6,7]. mEGF-PEG-PEI_25_ (83 nmol PEI) was mixed with SPDP (664 nmol in 100% ethanol) under argon. After 3 h at room temperature the mixture of about 2 ml was loaded on a gel filtration column (Sephadex G25 superfine; HR10/30; 20mM HEPES [pH 7.1], 0.5M NaCl; Amersham Biosciences). The purified PDP-functionalized conjugate (5 ml) containing 309 nmol of PDP was concentrated to 1.5 ml by speed vac. For the reaction with Melittin 464 nmol of Mel was weighed out and dissolved in 0.5 ml of 0.5 M NaCl, 100mM HEPES [pH 7.4] degassed with argon. Both components were mixed under argon. After 20 h at room temperature mEGF-PEG-PEI_25_-Mel was purified by gel filtration. To gel filtrate the conjugate, a Superdex 75 prep grade column 10/30, conditioned with PEI25br (10 mg PEI25/60 ml gel material) was used. After dialysis overnight (MWCO 14000; Visking type 27/32; Roth, Karlsruhe, Germany) against HBS 6 ml of mEGF-PEG-PEI_25_-Mel conjugate were obtained; these contained 66 nmol PEI (1.64 mg), 350 nmol Mel, and 70 nmol EGF.

### A431 Xenograft Treatment

Two million A431 cells were implanted subcutaneously into the left flanks of 15 nude CD-1 mice. When palpable tumors (average size 10.1 mm^3^) had developed, animals were divided into three groups of five animals. The complexes were delivered by intra-tumoral injection twice per day for 6 d. Five mice received poly IC-Melittin-PEI_25_-PEG-EGF (poly IC-MPPE) complex dissolved in HBG buffer at 0.1 μg poly IC/μl buffer (the dose of poly IC was 15 μg/injection or 30 μg/d), and five others received equivalent doses of MPPE complexes only (no poly IC). Remaining animals did not receive any treatment. Length *(a)* and width *(b)* of the tumors were measured daily with caliper and the tumor size was calculated as *ab^2^/2*. Control animals were sacrificed at day 33 after treatment initiation. Poly IC treated animals were left for follow up study to detect recurrence of the tumors.

### MDA-MB-468 Xenograft Treatment

Two million MDA-MB-468 cells were implanted over the mammary fat pad of 15 SCID/non-obese diabetic (NOD) mice. When palpable tumors (average size 9.8 mm^3^) had developed, animals were divided into three groups of five animals each. The complexes were delivered by intra-tumoral injection once per day for 6 d. Five mice received (poly IC)MPPE complex dissolved in HBG buffer at 0.1 μg poly IC/μl buffer (the dose of poly IC was 20 μg/d), and five others received an equivalent doses of MPPE complexes only (no poly IC). Tumor size was calculated as above. Control animals were sacrificed at day 33 after treatment initiation. Poly IC-treated animals were left for follow-up study to detect recurrence of the tumors.

## Results

### Fast and Selective Killing of EGFR Over-Expressing GBM Cells In Vitro

**Figure 1:**
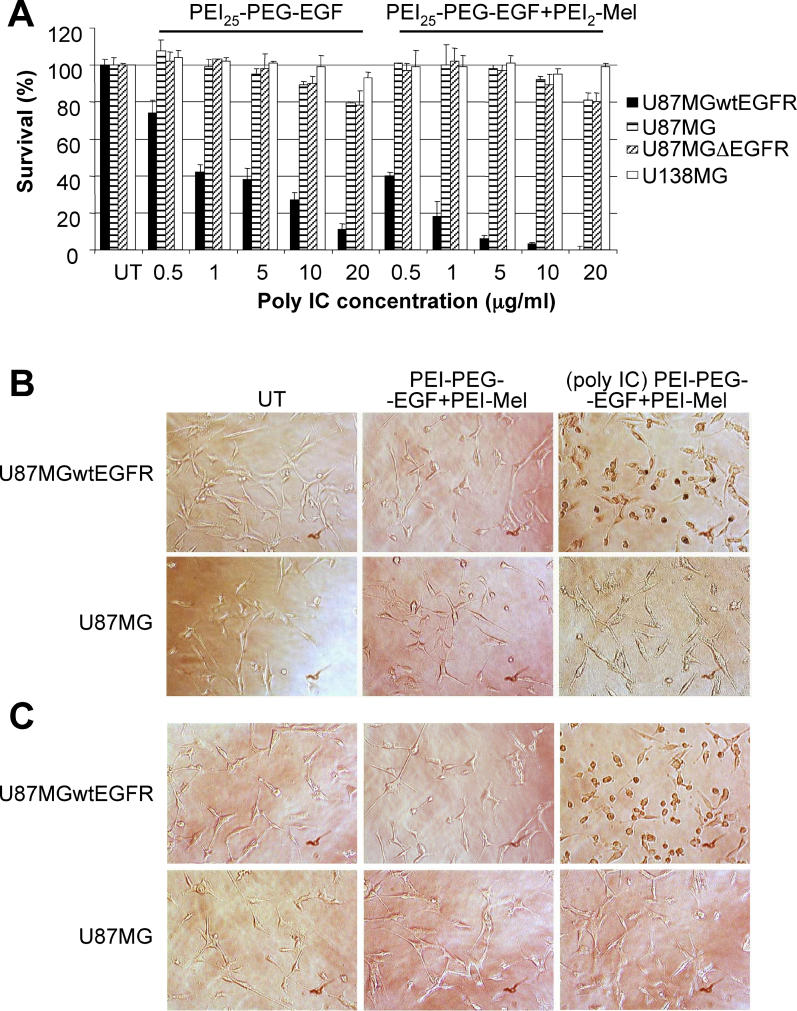
(poly IC)PEI-PEG-EGF(+PEI-Mel) Complexes Selectively Kill U87MGwtEGFR Cells (A) Cells were seeded in duplicate onto a 96-well plate at a density of 5,000 cells in 0.2 ml of medium per well and grown overnight. Cells were then transfected as described [6,9] with poly IC at the indicated concentrations using either PEI-PEG-EGF or PEI-PEG-EGF+PEI-Mel (w/w ratio PEI-PEG-EGF:PEI-Mel = 1:10) complexes. Viability was measured by the CellTiter-Glo Luminescent Cell Viability Assay (Promega) according to the manufacturer's instructions, at 1 h after transfection. (B and C) Fast induction of apoptosis by (poly IC)PEI-PEG-EGF+PEI-Mel complexes. Apoptotic death was detected 1 h after transfection by Annexin (B) and TUNEL (C) assays as described in Methods.

The PEI_25_-PEG-EGF complexes efficiently delivered poly IC, killing up to 85% of U87MGwtEGFR cells, which over-express EGFR (∼1 × 10^6^ receptors [10]), within 1 h of transfection (Figure 1A). At poly IC concentrations of up to 10 μg/ml, no significant effect was observed on the parental U87MG cells, which express 100,000 of EGFR per cell [10], on cells that over-express the mutated Δ(2–7)EGFR (U87MGΔEGFR), or on glioma cells lacking the EGFR (U138MG [11]) (Figure 1A). At the high concentration of 20 μg/ml poly IC, the survival of U87MG and U87MGΔEGFR cells, was inhibited by 20%. U138MG cells, which completely lack EGFR, were not inhibited at all. The killing effect on U87MGwtEGFR cells was enhanced 8- to 10-fold when PEI_25_-PEG-EGF was partially replaced with the polyethylenimine (2 kDa)-Melittin conjugate PEI_2_-Melittin (Figure 1A), and more than 95% of U87MGwtEGFR died within an hour of transfection, again, with no effect on the other glioma cell lines (Figure 1A). Melittin is a bee venom peptide that facilitates the release of nucleic acids from the endosome into the cytoplasm [9,12], thus enhancing the release of poly IC from the endocytosed vesicle. No significant toxic effect of the complexes without poly IC on any of the cell lines was detected (Figure S1). Annexin V and TUNEL staining showed that the majority (70%–90%) of the U87MGwtEGFR cells died by apoptosis within 1 h of transfection (Figure 1B and 1C). Although fast apoptotic death is not common, it has occasionally been detected in other cell lines where, like here, a number of pro-apoptotic pathways are activated simultaneously [13,14].

### Distribution of the Formulated Poly IC in the Cells

**Figure 2:**
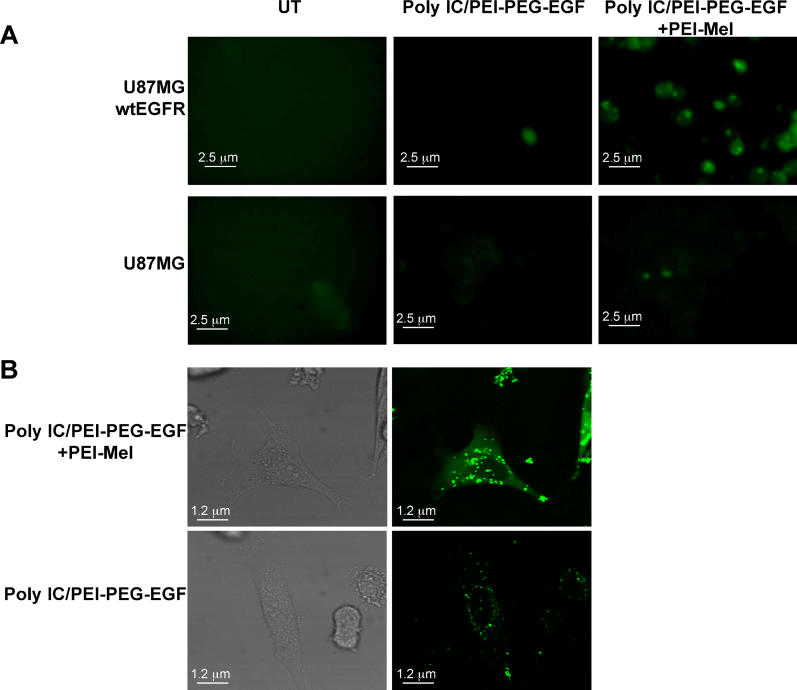
Distribution of the Complexes Cells were seeded into 6-cm plates at a density of 300,000 cells in 2 ml of medium per plate and grown overnight. Cells were then transfected with the fluorescently labeled poly IC (5 μg/ml) using the indicated conjugates. After 4 h, cells were analyzed by fluorescent microscopy (Methods) for selectivity of the transfection (A) and intracellular distribution of the complex in the U87MGwtEGFR cells (B).

In order to verify selective entrance of the complexes into the target U87MGwtEGFR cells and its release from endosomes fluorescent labeling of poly IC was performed (Methods). U87MGwtEGFR and U87MG cells were transfected with the labeled poly IC/PEI-PEG-EGF complex either in presence or absence of PEI-Mel (Figure 2). Figure 2A shows efficient transfection of the target U87MGwtEGFR cells and virtually no signal in U87MG cells. To examine the intracellular distribution, U87MGwtEGFR cells incubated with fluorescently labeled poly IC/PEI-PEG_25_-EGF or poly IC/PEI-PEG_25_-EGF+PEI-Mel complexes, were washed, and viewed live to rule out fixation artifacts, using confocal microscopy (Figure 2B). After 4 h of incubation, poly IC/PEI-PEG_25_-EGF complexes appeared in a punctate intracellular pattern, suggesting entrapment within vesicles (Figure 2B). In contrast, poly IC/PEI-PEG_25_-EGF+PEI-Mel complexes showed fluorescence dispersed throughout the cytoplasm (Figure 2B). The cytoplasmic fluorescence of the Melittin-containing complexes suggests that Melittin indeed facilitated release of the complex into the cytoplasm, by lysis of intracellular vesicles.

### Involvement of PKR in Cell Death

**Figure 3:**
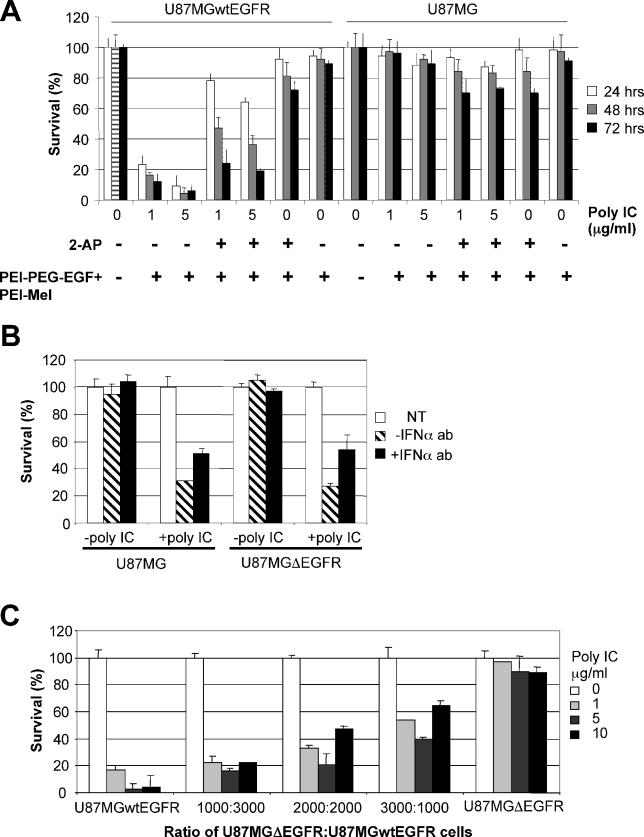
Cell Killing Mechanisms (A) Protection of the cells by 2-AP. Cells were grown as in Figure 1A. Cells were then transfected with poly IC at the indicated concentrations using PEI-PEG-EGF+PEI-Mel complexes. Where indicated, 2-AP (5 mM) was added 18 h before transfection and the medium was replaced every 24 h with medium containing fresh 2-AP. Viability was measured by the Methylene Blue assay [8]. (B) In vitro bystander effect. U87MGwtEGFR cells were grown and transfected as described in Methods. U87MG and U87MGΔEGFR “indicator” cells were grown in duplicates in 96-well plates. Medium of the “indicator cells” was then partially replaced by the medium collected from the transfected (**+poly IC**) or untransfected (**-poly IC**) U87MGwtEGFR cells (Methods). Where indicated the medium was preincubated with neutralizing polyclonal anti IFNα antibody. In **NT** samples, medium was not replaced. (C) A total of 4,000 U87MGwtEGFR and U87MGΔEGFR cells were seeded in duplicate onto a 96-well plate at the indicated ratios and grown overnight. Cells were then transfected with poly IC at the indicated concentrations using PEI-PEG-EGF+PEI-Mel conjugates. Cell survival was measured by the Methylene Blue assay 96 h after transfection.

The fast killing effect of the complexes was significantly inhibited by 2-aminopurine (2-AP), a potent inhibitor of PKR [15], suggesting that this effect is largely mediated by PKR (Figure 3A). At later time points, 48 and 72 h after transfection, the protective effect of 2-AP was significantly less pronounced, suggesting that additional cell killing mechanisms were activated.

### In Vitro Bystander Effect

We next investigated whether the poly IC-transfected cells exerted a bystander effect on untransfected cells (Methods). Growth medium from poly IC-transfected U87MGwtEGFR cells inhibited the growth of U87MG and U87MGΔEGFR cells (Figure 3B), which are themselves insensitive to the (poly IC)PEI-PEG-EGF+PEI-Mel complex (Figure 1A). The bystander effect was less pronounced when U87MGwtEGFR cells were transfected with targeted poly IC at higher concentrations (Figure S2), probably because the transfected cells died before they could exert the full effect. The bystander effect was further confirmed by co-culture of U87MGwtEGFR and U87MGΔEGFR cells in the same wells (Figure 3C). A large fraction of the U87MGΔEGFR cells were killed, although they are themselves almost insensitive to the poly IC complex.

**Figure 4:**
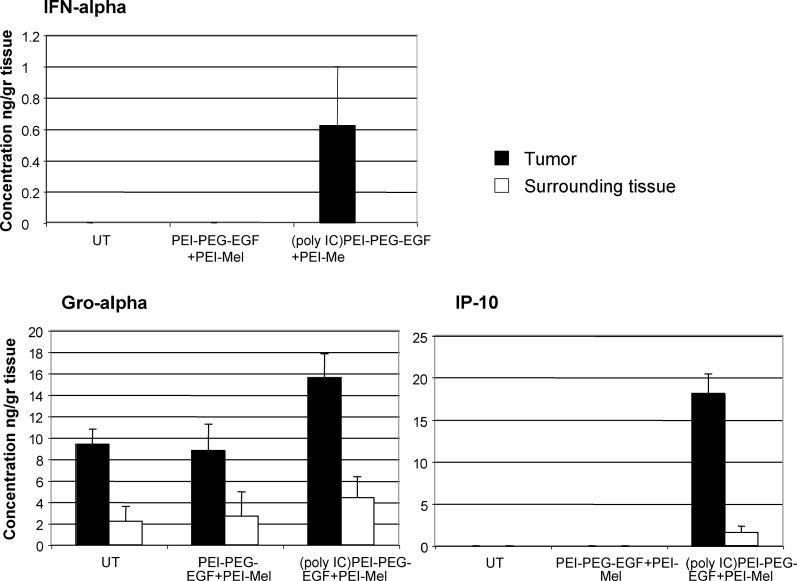
Targeted Poly IC Induces Expression of Cytokines For this assay, 10,000 U87MGwtEGFR cells were implanted into the brain of nude mice. After 15 d, poly IC was injected with Alzet micropumps for 24 h at 0.8 μg/hr. After 48 h, animals were sacrificed and the brains were extracted. Tumors (average weight 17.6 mg) and surrounding brain tissue (average weight 253 mg) were resuspended in Tris-HCl buffer (100 mM Tris, [pH 8.1] with 1% Triton X-100) at 1:10 w/v. Samples were homogenized on ice by sonication, triturated through 19 gauge needles and spun at 20,000 × g. Protease inhibitors were added to supernatant, which was then subjected to cytokine-specific ELISA.

Double-stranded RNA is known to induce the expression of interferons and other anti-proliferative cytokines. To investigate whether the bystander effect was caused by interferon, we incubated medium from transfected U87MGwtEGFR cells with neutralizing IFNα antibody. This pre-incubation reduced the bystander effect on all cell lines by 20%–30% (Figure 3B), indicating that IFN-α was indeed responsible for part of the bystander effect. The partial reduction in the bystander effect suggests that additional cytokines, other than IFN-α, were also expressed. Using ELISA (IBL, Hamburg, Germany), we confirmed the presence of IFNα in growth medium from poly IC-transfected cells. Up to 4 pg/ml IFNα were generated in the medium of 500,000 U87MGwtEGFR cells transfected with 2.5 μg/ml of poly IC, but not in the medium of U87MG or U87MGΔEGFR cells treated with the same complex. No IFN-α was detected in medium from cells transfected with poly IC at higher concentrations, suggesting that these cells were killed before they could secrete the cytokine. IFN-α was also generated in vivo (> 600 pg/g of tumor tissue), specifically in U87MGwtEGFR tumor xenografts (Figure 4). Using a Human Cytokine Microarray (RayBiotech, Norcross, Georgia, United States), we sought to identify additional cytokines in the medium of poly IC-transfected cells. Both GROα [16] and IP-10 [17] were detected. The production of IP-10 and Gro-alpha at high concentrations in vitro and selectively in tumors in vivo, was confirmed by cytokine specific ELISAs (Figure 4). These cytokines are chemokines responsible for the recruitment of T-cells to area of expression [16,17].

### Formulated Poly IC Eliminates Intracranial GBM Models in Mice

**Figure 5:**
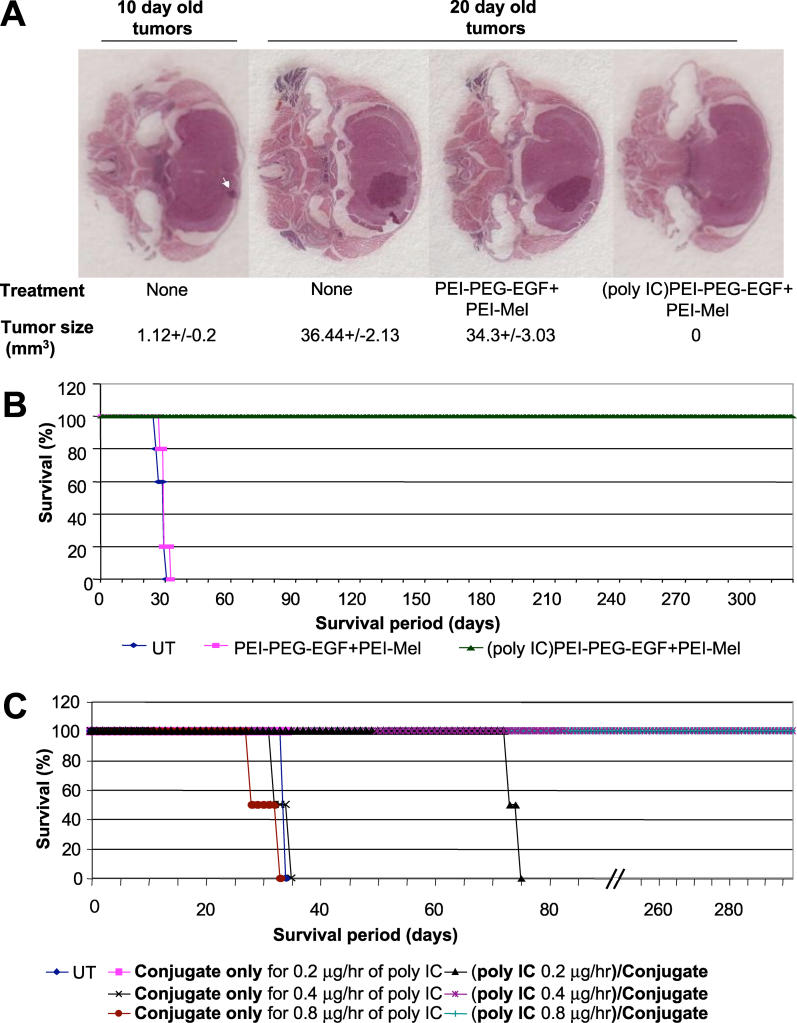
Targeted Poly IC Eliminates Intracranial GBM Models (A and B) (poly IC)PEI-PEG-EGF+PEI-Mel complexes eliminate U87MGwtEGFR xenografts. Intracranial U87MGwtEGFR tumors were established and treated with formulated poly IC as described in Methods. (A) Sizes of the tumors before and after the treatment. (B) Survival of the animals (Methods). (C) In vivo bystander effect. A mixture of 5,000 U87MGwtEGFR and 5,000 U87MGΔEGFR cells was implanted into the brain of nude mice as described in Methods. The mixed tumors were treated with formulated poly IC (**poly IC-/conjugate)** at the indicated doses, and control animals received equivalent doses of PEI-PEG-EGF+PEI-Mel complexes without poly IC (**Conjugate only**). Survival of the animals was analyzed as above. Mice receiving 0.2μg/h of poly IC die up to day 75, whereas those receiving 0.4 μg poly IC/h and 0.8 μg poly IC/h are still alive.

**Figure 6:**
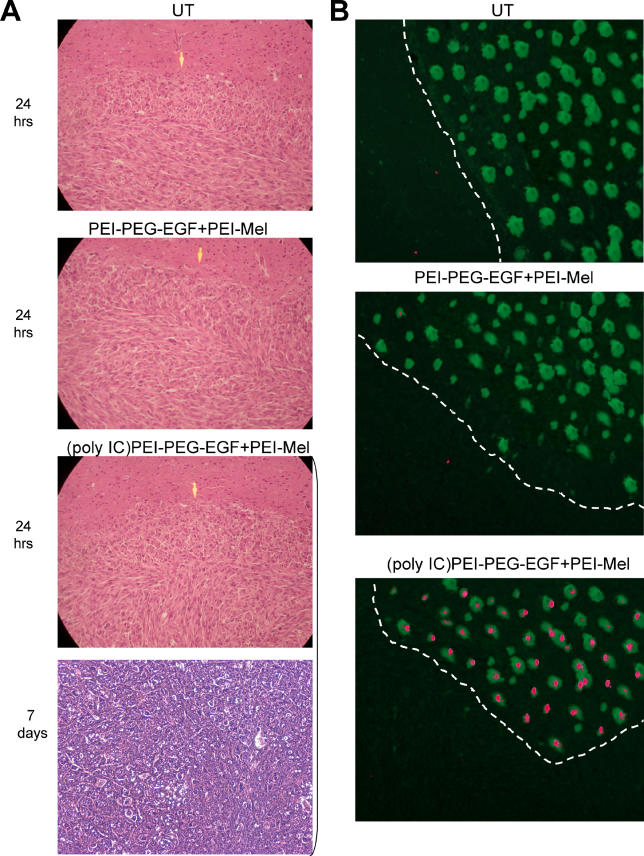
In Vivo Selectivity of the Approach (A) Pathological analysis of the brains. Intracranial tumors were established and treated as described in Methods. After 24 h, animals were sacrificed and ultrathin slices of the brains were prepared. Slices were stained with H & E and analyzed by light microscopy (20× magnification) for development of pathological signs and immune cell infiltration (yellow arrow). Bottom panel shows the example of slices from the brains treated with (poly IC)PEI-PEG-EGF+PEI-Mel in the experiment described in Figure 5A (7 d post-treatment). (B) Poly IC induces apoptosis in intracranial xenografts. Intracranial tumors were established and treated as described in Methods. Apoptotic death was detected using Cell Death Detection kit-TMR Red (Methods). White dashed lines represent borders of the tumors.

These encouraging results led us to test the EGFR-targeted poly IC strategy in vivo*.* Ten thousand U87MGwtEGFR cells were implanted into the brains of nude mice as described (Methods) and the tumors left to grow for 10 d. During this period, visible tumors developed (Figure 5A). On day 10 after cell implantation, (poly IC)PEI-PEG-EGF+PEI-Mel complexes were delivered directly into the tumors at a constant rate for 3 d, using Alzet osmotic micropumps. On day 20 after cell implantation (7 d after the end of treatment), the tumors had disappeared completely from (poly IC)PEI-PEG-EGF+PEI-Mel-treated animals, while the tumors continued to grow in untreated animals until reaching 36.44 mm^3^ (Figure 5A). Animals that were treated with PEI-PEG-EGF+PEI-Mel alone (no poly IC), survived for no longer than 32 d, as did untreated animals (Figure 5B). In contrast, the (poly IC)PEI-PEG-EGF+PEI-Mel-treated animals are still alive on the day of submission of this manuscript (more than a year), and show no signs of increased intracranial pressure. When Melittin was omitted, the mice died from GBM 57–59 d after cell implantation (unpublished data). Histopathologic examination of the brains of the poly IC-treated mice at day 7 after the end of treatment did not reveal any residual tumors (Figures 5A and 6A), whereas the control animals had large tumors (Figures 5A and 6A). Pathological analysis of the brains at 24 h after treatment initiation showed increased gliosis in the brains of the animals of all experimental groups (Figure 6A). This was probably caused by the growing tumors in the brains. We also detected low infiltration of macrophages into the tumors in all groups (Figure 6A). None of the animals treated with poly IC showed any additional histopathological signs of toxicity or brain tissue damage either at 24 h after treatment initiation or at 7 d after the end of the treatment (Figures 5A and 6A). Similarly, we did not detect any significant increase in infiltration of immune cells into poly IC treated tumors as compared with the untreated tumors (Figure 6A).

### Bystander Effect In Vivo

In the clinical setting, some GBM cells over-express wt EGFR, whereas neighboring cells express lower levels of EGFR or over-express truncated EGFR (ΔEGFR) [2]. As described above, in culture the EGFR-targeted poly IC-transfected cells induced the killing of neighboring, untransfected cells (Figure 3B and 3C). We therefore examined bystander killing in vivo (Methods). We implanted a mixture of 5000 U87MGwtEGFR and 5000 U87MGΔEGFR cells in mice brains (Figure 5C). The mixed tumors that developed 10 d later were treated with the targeted poly IC at various doses (Figure 5C). Control animals survived for no more than 34 d, while mice that received the lowest dose of poly IC complex (0.2 μg/h) survived more than 2 times longer. Animals that received higher doses of formulated poly IC (0.4 μg/h and 0.8 μg/h) are alive and well on the day of submission of this paper (day 297+). This very encouraging result is probably due to the strong bystander effect induced by the slow delivery of the formulated poly IC. Elimination of the mixed tumor was not caused by immune reaction since we did not detect increased infiltration of immune cells into the poly IC treated tumors (Figure 6A).

### Formulated Poly IC Induces Apoptosis in U87MGwtEGFR Cells In Vivo

We next examined the mode of U87MGwtEGFR cell death induced by the targeted poly IC in vivo. U87MGwtEGFR cells were injected into mice and the established tumors were treated with formulated poly IC. EGFR expression was monitored with FITC-conjugated EGFR antibody (green fluorescence). Apoptosis was monitored with the TMR Red Cell Death Kit (Roche). Apoptosis occurred exclusively in cells over-expressing the EGFR (Figure 6B). No significant red signal was observed in the surrounding tissue, demonstrating the high selectivity and low toxicity of the treatment regimen.

### Effect of Formulated Poly IC on 15-d-Old Intracranial Xenografts

Although 10-d tumors used in the survival experiments are clinically relevant to newly diagnosed GBM in humans (R. Catane and R. Pfeffer, Division of Neuro-oncology, Tel Hashomer/Sheba Hospital, personal communication), we examined whether formulated poly IC is effective against much larger tumors (Figure 5A). For this purpose we synthesized Mel-PEI_25_-PEG-EGF (MPPE, Methods). Although not observed, there is a possibility of PEI-Mel dissociation from (poly IC)PEI-PEG-EGF+PEI-Mel complexes. This may result in decreased release of poly IC to cytoplasm. When both Melittin and EGF are covalently bound to the same PEI molecule, the vector should be more efficient and even less toxic, because Melittin is now covalently linked to the complex, and therefore there is no possibility of PEI-Mel leakage. It was also observed that PEI_25_ conjugates are significantly smaller in size than PEI_2_ or PEI_2_+PEI_25_ conjugates (M.O., unpublished data), leading to higher in vivo transfection efficiency.

**Figure 7:**
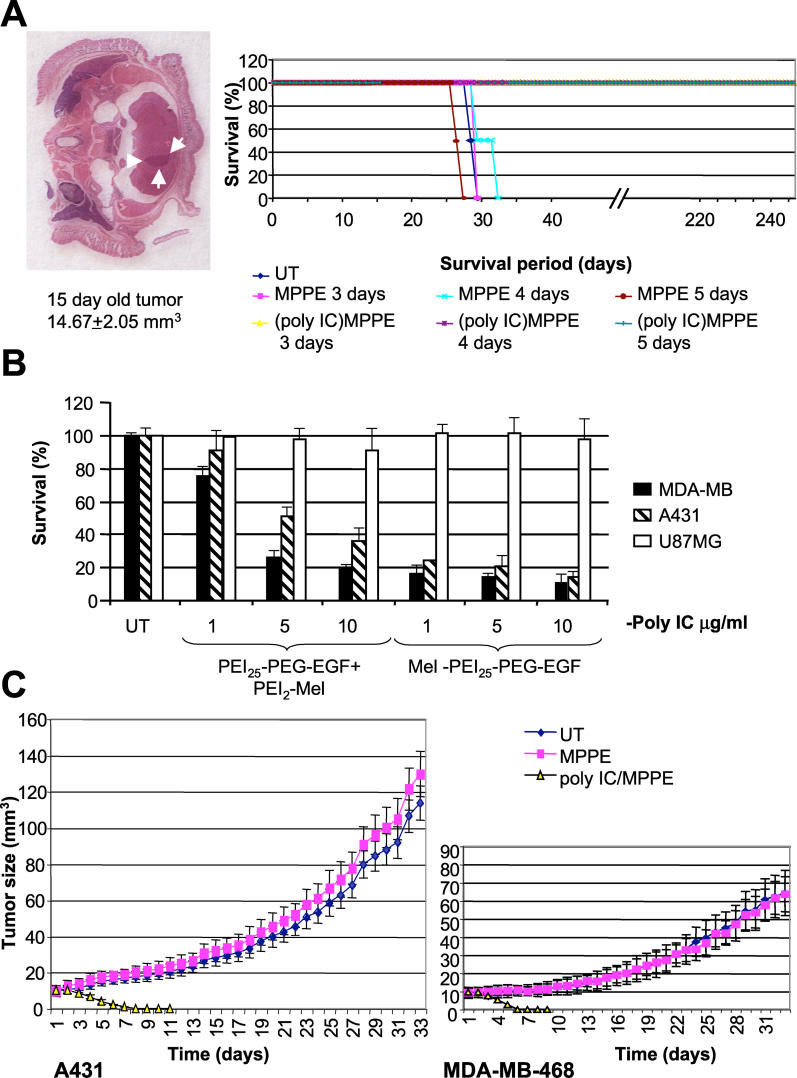
Targeted Poly IC Eliminates Three Types of EGFR Over-Expressing Tumors (A) (poly IC)Mel-PEI-PEG-EGF (MPPE) complexes prolong survival of mice bearing large intracranial U87MGwtEGFR xenografts. Cells were implanted into the brains of 16 mice. 15 d later, two animals were sacrificed to measure the tumors (Methods). Other animals received the indicated treatments. The daily doses of complexes were similar to the doses in Figure 3A and 3B. Survival of the animals was analyzed as above. (B) Formulated poly IC selectively kills A431 and MDA-MB-468 cells. Cells were seeded in duplicate onto a 96-well plate at a density of 5,000 cells in 0.2 ml of medium per well and grown overnight. Cells were then transfected as described [6,9] with poly IC at the indicated concentrations using either MPPE or PEI-PEG-EGF+PEI-Mel. Cell survival was measured by the Methylene blue assay at 48 h after transfection. (C) (poly IC)MPPE complexes eliminate A431 and MDA-MB-468 xenografts in mice. A431 and MDA-MB-468 tumors were established and treated with formulated poly IC as described in Methods. Tumors were measured daily. Control animals were euthanized at day 33 after treatment initiation. Poly IC treated mice were kept alive to detect possible late recurrence of the tumors.

In order to obtain larger tumors, the intracranially implanted 10,000 U87MGwtEGFR cells were left to grow for 15 d. The resulted tumors were 13–15 times bigger than the 10-d-old tumors (Figures 5A and 7A). (poly IC)Mel-PEI-PEG-EGF and (poly IC)MPPE complexes were delivered directly into the tumors at a constant rate for 3, 4, and 5 d, using Alzet osmotic micropumps. Animals that were treated with MPPE alone (no poly IC), survived for no longer than 33 d, as did untreated animals (Figure 7A). In contrast, the (poly IC)MPPE-treated animals in all groups are still alive on the day of submission of this manuscript (day 244 +), and show no symptoms of GBM.

### Formulated Poly IC Destroys Other EGFR Over-Expressing Cancers In Vitro and In Vivo

We next examined whether our strategy could be implemented for the treatment of other EGFR over-expressing cancers. We used the A431 (vulval carcinoma, expressing ∼2 × 10^6^ EGF receptors per cell [18]) and MDA-MB-468 (breast cancer, similar EGFR expression and EGFR responsiveness to A-431 cells [19]), both non-engineered cell lines. Figure 7B shows targeted poly IC induced killing of A431 and MDA-MB-468 cells, whereas the control U87MG cells remain unharmed. Figure 7B also demonstrates the enhanced efficacy of the all-in-one vector MPPE as compared to PEI-PEG-EGF+PEI-Mel combination formula.

Encouraged by these results, we examined the efficiency of our strategy on models of these cancers in vivo*.* A431 cells were implanted subcutaneously into nude mice as described (Methods) and the tumors left to grow for 10 d. During this period, tumors of average size 10.1 mm^3^ were developed (Figure 7C). After tumor establishment, (poly IC)MPPE complexes were injected directly into the tumors twice per day for 6 d at 15 μg of poly IC/mouse/injection (30 μg/d) (Methods). On day 10 after treatment initiation, the tumors had disappeared completely from (poly IC)MPPE treated animals, while the tumors continued to grow in untreated animals, reaching up to139 mm^3^ (Figure 7C).

MDA-MB-468 cells were implanted over mammary fat pads of female SCID mice and the tumors left to grow for 14 d. During this period, tumors of average size 9.7 mm^3^ developed (Figure 7C). (poly IC)MPPE complexes were injected directly into the tumors for 6 d. On day 8 after treatment initiation, the tumors had disappeared completely from (poly IC)MPPE animals, while the tumors continued to grow in untreated animals, reaching 63 mm^3^ (Figure 7C).

We did not detect recurrence of any of the tumors in the follow up study (day 42+ after treatment initiation).

## Discussion

Our results suggest that the EGFR-targeted delivery of poly IC can be implemented in the clinical treatment of GBM, for which current therapies are essentially ineffective. The therapeutic approach described here incorporates rapid and efficient killing of tumor cells by multiple mechanisms, a strong bystander effect, along with high selectivity. The localization of GBM in the central nervous system makes it an attractive candidate for local therapy by slow, constant, intra-tumoral delivery of the complexes described here (Figure 5). EGFR-targeted delivery of poly IC should be especially effective for the small tumors remaining after surgery, but could in principle be the first line of treatment, considering the results reported here. We have previously described an approach for GBM treatment using viral vectors to induce the in situ production of cancer-specific dsRNA within the cancer cell [8]. The EGFR-targeted synthetic dsRNA approach we describe now seems to be far superior, probably because we have achieved rapid delivery of a large dose of longer dsRNA into the cancer cell. This leads to a very fast response and induction of a bystander effect so that the tumor is demolished more rapidly than it can re-grow. Although many of the immune cells are still present in the nude mice we used, no significant immune reaction against the poly IC treated tumor was observed (Figure 6A). Most likely absence of the immune response is due to the fast elimination of the tumor. An immune reaction might be induced in patients following treatment that will take significantly longer periods of time. Nevertheless, expression of immunoactive IFN-α and T-cell chemokines, IP-10 and Gro-α selectively in the tumor (Figure 4) should drive the immune response specifically against GBM, reducing potential toxic effects on surrounding brain tissue.

To the best of our knowledge, the strategy described in this study has yielded the most effective treatment of EGFR over-expressing GBM reported so far, in an animal model. The therapeutic strategy described here differs significantly from other EGFR targeted agents aimed at GBM, such as erlotinib, gefitinib, [20] and anti-EGFR antibodies [21]. These agents inhibit the activity of the receptor and its downstream signaling. The response of GBM to gefitinib and erlotinib in GBM is weak [22]. Furthermore, the response of non-small-cell-lung cancer to either drug as a single agent is limited to tumors in which the EGFR harbor mutations in the kinase domain, where the EGFR is a survival element, and is usually transient [23]. Mutations (extracellular) in the EGFR receptor or frequent heterogeneity of GBM where only part of the cells over-express EGFR will result in only partial response to gefitinib and erlotinib as indeed observed in the recent clinical trials [22]. In the case of anti-EGFR therapy, the response seems not to be related to whether the EGFR is mutated but these drugs are also not life saving in patients with tumors involving EGFR [24]. The strategy described here is different from those currently implemented therapies because the EGFR is utilized to target a vehicle that delivers a strong pro-apoptotic molecule, namely dsRNA. Due to the strong bystander effect induced by the massive amount of dsRNA on neighboring cancer cells that were not targeted themselves, the therapy described here can eradicate EGFR over-expressing tumors even when only half of the cells over-express wt EGFR (Figure 5C). The bystander effect is mediated at least partially by IFN-α (Figure 3B). This cytokine is clinically used against various cancers including GBM. Since IFN-α kills mainly fast proliferating cells, normal cells should not be affected. Indeed, we did not detect any pathology in normal brain tissue during or after the therapy (Figure 6A).

Another important advantages of the therapy described here are the fast cell killing (Figure 1) and activation of several growth inhibitory pathways simultaneously (Figures 3A and 4). This form of therapy is likely to prevent and/or overcome a potential mutation in one of the anti-proliferative proteins and development of resistance to the therapy. This is especially relevant for cancers with deficient PKR activity [25], because poly IC can still induce cell death in a PKR independent manner (Figure 3A). We would like to suggest that if this strategy is translated successfully to the clinical setting it may indeed help patients with GBM. In fact, several neurosurgery teams are interested in implementing this therapy to clinic once all preclinical studies will be completed.

The elimination of EGFR over-expressing adenocarcinoma and breast cancer in vivo (Figure 7C) suggests that in principle this strategy can be applied to treat other cancers that over-express EGFR. It should be emphasized that there are a number of scenarios, other than GBM in which the local application of the EGFR targeted poly IC is advantageous, like head and neck cancer in which EGFR is over-expressed and local therapies are often used.

Finally, we would like to suggest that the strategy of ligand-guided delivery of dsRNA described here, can in principle, be applied to other cancers in which a particular receptor is over-expressed and undergoes endocytosis. Receptors that are over-expressed in many tumors and qualify as candidates for targeting poly(IC) include the transferrin receptor [26], the PDGF receptor [27] and the IGF-1 receptor [28].

## Supporting Information

Figure S1 (poly IC)PEI-PEG-EGF(+PEI-Mel) Complexes Selectively Kill U87MGwtEGFR Cells Cells were seeded onto a 96-well plate at a density of 5,000 cells in 0.2 ml of medium per well and grown overnight. Cells were then transfected as described [6,9] with poly IC at the indicated concentrations using either PEI-PEG-EGF or PEI-PEG-EGF+PEI-Mel (w/w ratio PEI-PEG-EGF:PEI-Mel = 1:10) complexes. The viability was measured by the CellTiter-Glo Luminescent Cell Viability Assay (Promega) according to the manufacturer's instructions at 1 h after transfection. Transfection with native PEI and Fugene6 (Roche) was used to control for non-specific effect of poly IC. HBS stands for transfection buffer alone [6].
(119 KB PDF).

Figure S2 Bystander Effect For this assay, 500,000 U87MGwtEGFR cells were seeded onto 6-cm plates, grown overnight in 2 ml of medium, and transfected with poly IC at the indicated concentrations using PEI-PEG-EGF+PEI-Mel complexes. Medium was collected at the indicated times after transfection (*x*-axis). U87MG and U87MGΔEGFR “indicator” cells were seeded in 96-well plates (4,000 cells/well) and grown overnight in 200 μl of medium. 100 μl of medium was then replaced by the medium collected from the transfected U87MGwtEGFR cells. In NR samples, medium was not replaced. Growth inhibition was examined 48 h after medium exchange.
(35 KB PDF).

**Patient Summary**

**Background**

Glioblastomas are the most frequent and the most aggressive human brain tumors. Scientists have learned more about these tumors in the past few years, but there are still very few treatment options besides surgery and radiation, and only 3% of patients survive longer than 5 years after diagnosis.

**Why Was This Study Done?**

Over half of glioblastomas have a particular characteristic: the cells that make up the tumor have 10–100 times more of a particular molecule called EGFR on their surface than the surrounding non-tumor cells. Taking advantage of that fact, the researchers devised a strategy that used EGFR as a target to deliver a treatment that they hoped would kill the tumor cells.

**What Did the Researchers Do and Find?**

All of the experiments were done with cultured cells, and some of them used mice in which tumors had been implanted. The researchers created a mix of chemicals that would latch on to the EGFR on the tumor cell surfaces, be taken up by the tumor cells, and once inside the tumor cell would instruct it to commit suicide. They found that this approach worked in cultured tumor cells with lots of EGFR on their surfaces, and in mice that had brain tumors comprised of such cells. They found that the treatment was quite specific (that is, it killed the tumor cells and not the surrounding normal brain tissue) and effective (all of the tumor cells seemed to have been killed and the tumors didn't grow back).

**What Does This Mean?**

These results are encouraging. Many questions need to be answered before we know whether this approach or a similar one will be practical and efficient against human glioblastomas. Given the lack of current options, it certainly seems worth pursuing, and the researchers are currently preparing essential preclinical studies in which a clinical-grade drug will be prepared, and its toxicity, distribution to various organs, and other important analytical studies performed. At present the authors examine in more detail the toxicity profile of the poly IC complexed to the EGF receptor targeting vector.

**Where Can I Find More Information Online?**

The following Web sites provide information on brain cancer and glioblastomas.

National Brain Tumor Foundation in the US:

http://www.braintumor.org/

Cancer BACUP pages on brain cancer:

http://www.cancerbacup.org.uk/Cancertype/Brain

The Brain Tumor Society:

http://www.tbts.org/

NCI information pages on brain tumors:

http://www.cancer.gov/cancertopics/types/brain/

## Abbreviations

2-AP: 2-aminopurine
dsRNA: double-stranded RNA
DTT: dithiothreitol
EGF: epidermal growth factor
EGFR: epidermal growth factor receptor
GBM: glioblastoma multiforme
mEGF: recombinant mouse EGF
MPPE: melittin-polyethyleneimine-polyethyleneglycol-EGF
NHS-PEG-MAL: N-hydroxysuccinimide-PEG maleimido
PEI: polyethylenimine
PEI_25_-PEG-EGF: polyethylenimine-polyethyleneglycol-EGF
PKR: double-stranded RNA-dependent protein kinase
poly IC: polyinosine-cytosine
SCID: severe combined immunodeficiency
