# Correction: EGF Receptor-Targeted Synthetic Double-Stranded RNA Eliminates Glioblastoma, Breast Cancer, and Adenocarcinoma Tumors in Mice

**DOI:** 10.1371/journal.pmed.0040266

**Published:** 2007-08-28

**Authors:** Alexei Shir, Manfred Ogris, Ernst Wagner, Alexander Levitzki

Correction for:

Shir A, Ogris M, Wagner E, Levitzki A (2006) EGF Receptor-Targeted Synthetic Double-Stranded RNA Eliminates Glioblastoma, Breast Cancer, and Adenocarcinoma Tumors in Mice. PLoS Med 3(1): e6. doi:10.1371/journal.pmed.0030006

After this article was published, we were alerted by a reader to a potential problem with Figure 6B. Subsequent investigation determined that this figure is indeed incorrect.

**Figure 6:**
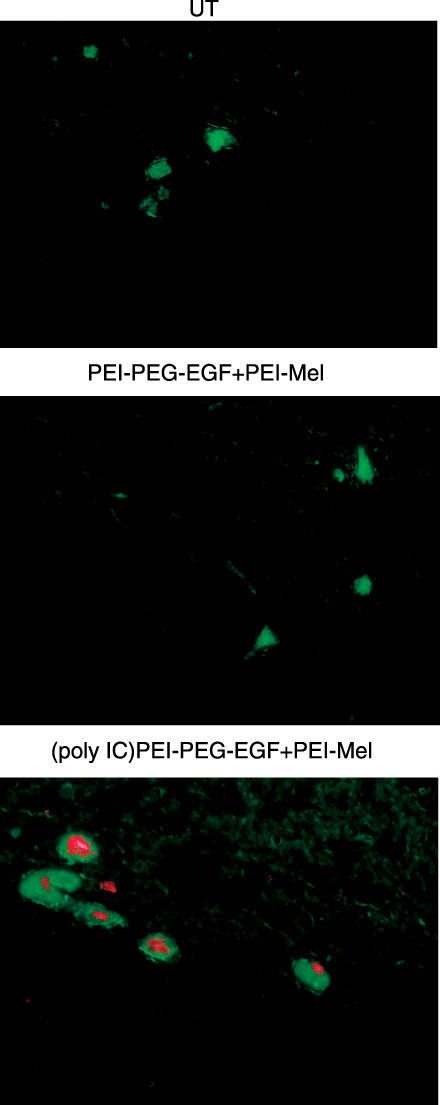
In Vivo Selectivity of the Approach (B) Poly IC induces apoptosis in intracranial xenografts. Intracranial tumors were established and treated as described in Methods. Apoptotic death was detected using Cell Death Detection kit-TMR Red (Methods).

The two lower panels come from the same tumor rather than different tumors as indicated in the legend. The authors have provided the correct figure, substituting images of samples taken from different tumors from the same experiment.

The authors regret this error and are willing to provide all reagents necessary to repeat the experiment in question to interested scientists. Requests for reagents should be addressed directly to the corresponding author at levitzki@vms.huji.ac.il.

