# Supplementary material for: EGF Receptor-Targeted Synthetic Double-Stranded RNA Eliminates Glioblastoma, Breast Cancer, and Adenocarcinoma Tumors in Mice
Source: PLoS Med. 2005 Dec 6;3(1):e6. doi: 10.1371/journal.pmed.0030006 (PMC1298941; doi:10.1371/journal.pmed.0030006)
Supplement: Supplementary file 1 [file pmed.0030006.sg001.pdf]

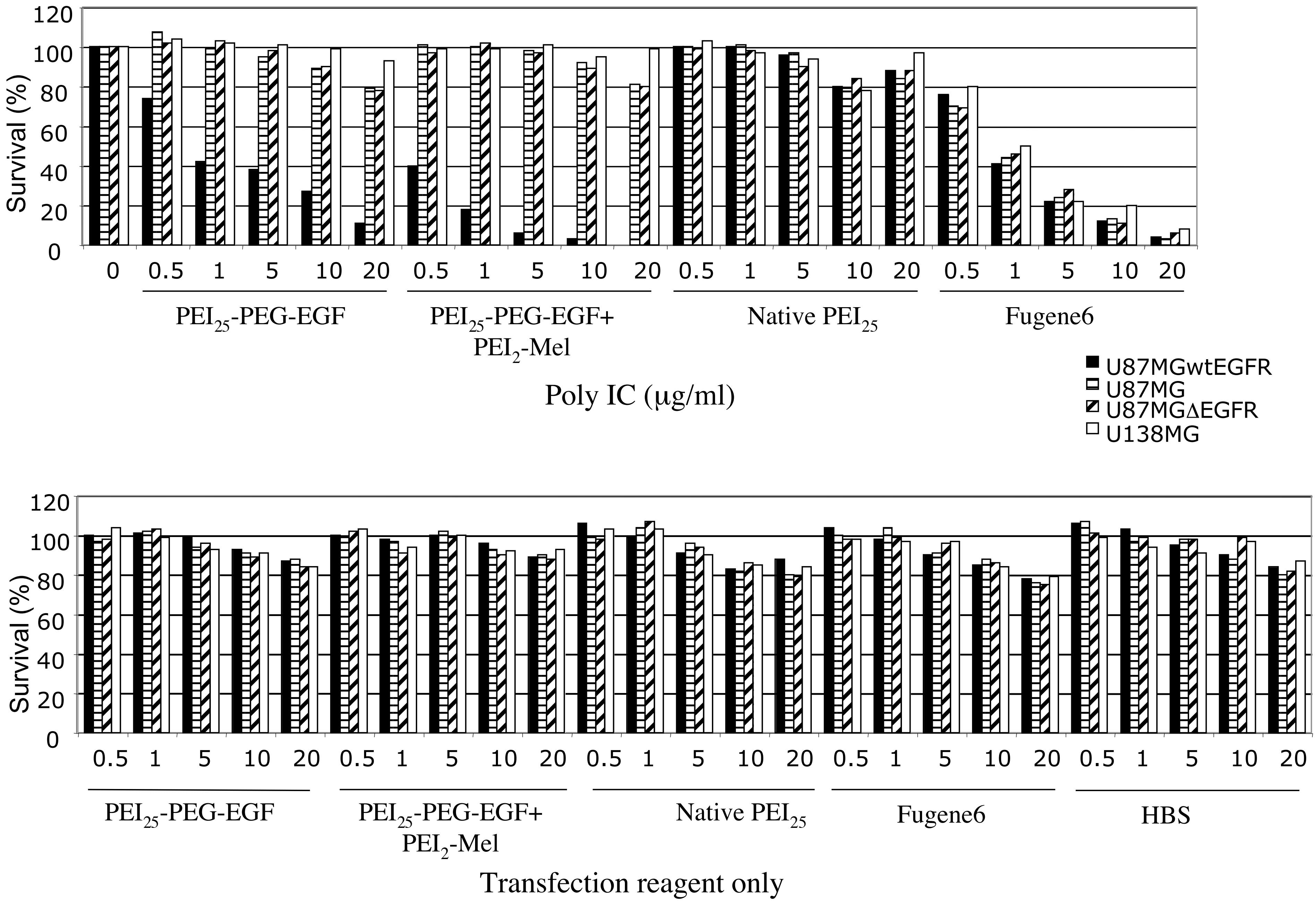

120
100
80
60
40
20
0
Survival (%)
0
0.5
1
5
10
20
PEI25-PEG-EGF
PEI25-PEG-EGF+
PEI2-Mel
Native PEI25
Fugene6
Poly IC (μg/ml)
U87MGwtEGFR
U87MG
U87MGΔEGFR
U138MG
HBS
Transfection reagent only
