# Supplementary material for: EGF Receptor-Targeted Synthetic Double-Stranded RNA Eliminates Glioblastoma, Breast Cancer, and Adenocarcinoma Tumors in Mice
Source: PLoS Med. 2005 Dec 6;3(1):e6. doi: 10.1371/journal.pmed.0030006 (PMC1298941; doi:10.1371/journal.pmed.0030006)
Supplement: Supplementary file 2 [file pmed.0030006.sg002.pdf]

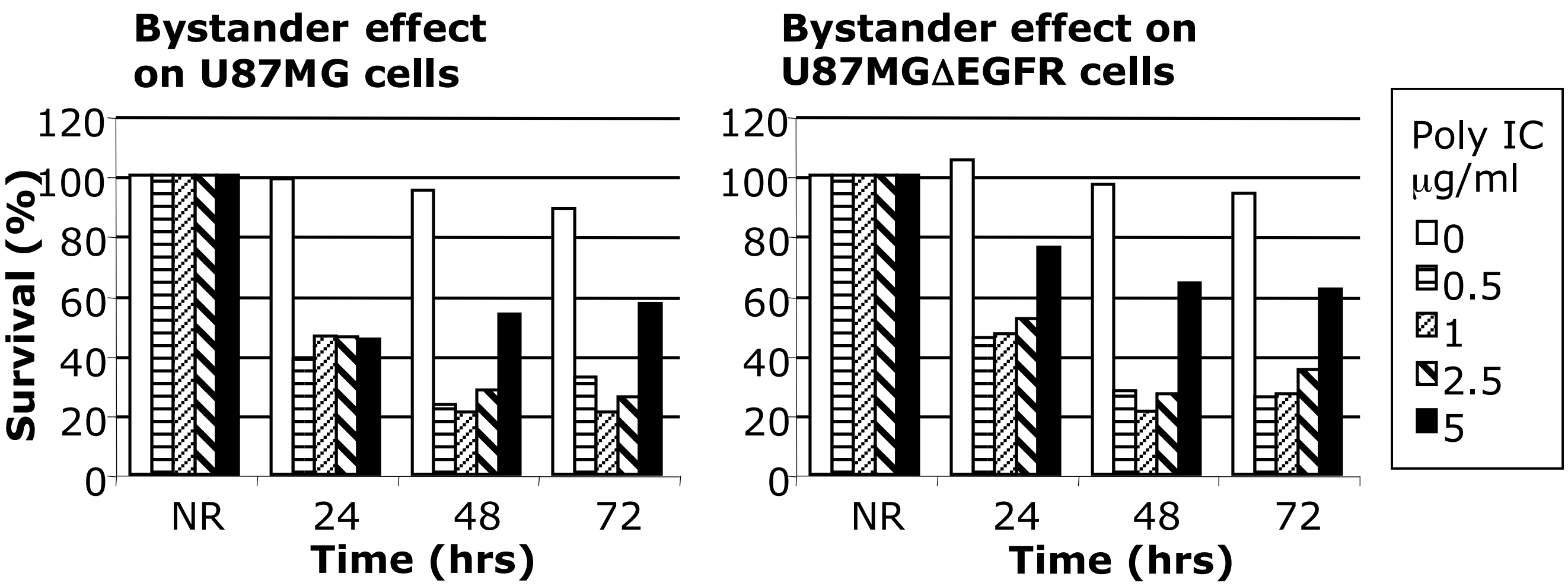

Bystander effect on U87MG cells
Bystander effect on U87MGΔEGFR cells
Survival (%)
120
100
80
60
40
20
0
NR
24
48
72
Time (hrs)
Poly IC μg/ml
0
0.5
1
2.5
5
